# A case of bilateral visual loss after spinal cord surgery

**DOI:** 10.11604/pamj.2016.23.119.8443

**Published:** 2016-03-24

**Authors:** Jemal Shifa, Worknehe Abebe, Negussie Bekele, Dereje Habte

**Affiliations:** 1 Department of Surgery, Faculty of Medicine, University of Botswana, Gaborone, Botswana; 2 Department of Anesthesiology, Faculty of Medicine, University of Botswana, Gaborone, Botswana; 3 Management Sciences for Health, HEAL TB Project, Addis Ababa, Ethiopia

**Keywords:** Visual loss, anesthesia, complication, anemia

## Abstract

Visual loss is a rare but potentially devastating postoperative complication of prone spinal surgery with a reported incidence of 0.017 to 0.1 percent. We present a case of post-operative bilateral visual loss in a patient who had a laminectomy in prone position under general anesthesia. A 17-year-old male patient with large syringomyelia extending from C2 – T2 level had a surgical procedure done under general anesthesia (GA) in prone position that lasted four hours. After the surgical procedure, the patient presented to the Ophthalmology Clinic of Princess Marina Hospital, with a complaint of visual loss of the right eye followed by left, of one week duration. The patient never had a visual impairment in the past. Physical examination, fundal examination and CT scan revealed no primary cause for the visual loss. In this patient the absence of any finding in the optic disc and the retina and the normal CT scan, suggests that the most likely diagnosis is posterior ischemic optic neuropathy. The patient was treated with prednisolone tablet daily and showed mild improvement in vision.

## Introduction

Visual loss is a rare but potentially devastating postoperative complication of prone spinal surgery with a reported incidence of 0.017 to 0.1 percent [1]. The major causes of visual loss in these patient populations include ischemic optic neuropathy (ION), central retinal artery occlusion (CRAO), and retinal vein occlusion (RVO) [1]. Ischemic optic neuropathy is the most common cause of postoperative visual loss. The American Society of Anesthesiologists (ASA) closed claims database reports that 81% of postoperative visual loss is diagnosed as ischemic optic neuropathy and the remainder as retinal artery thrombosis. Sixty-seven percent of all the reported cases of postoperative visual loss occurred after spinal surgery in the prone position [1]. In this report, we present a case of post-operative bilateral visual loss in a patient who had a laminectomy in prone position under general anesthesia.

## Patient and observation

A 17-year-old male patient presented to the General Outpatient Department of Princess Marina Hospital (a referral hospital in Gaborone, Botswana) with weakness of all the limbs and walking with difficulty of two weeks duration. He is a student at secondary level who neither had any current complaint nor past history of visual impairment. He weighed 46 kg and was 160 cm tall, with BMI of 17.9. He had no history of hypertension, diabetes mellitus, visual impairment or cigarette smoking. Neurological examination showed weakness of both upper and lower limbs. Magnetic resonance imaging (MRI) of the spine revealed large syringomyelia extending from C2 – T2 level. There was no associated brain lesion.

He was referred to a medical center in Johannesburg-South Africa for further evaluation and surgical management. Surgery was done in South Africa Medical Center was reported to be C5-C6 laminectomy with syringo-subarachnoid shunt. The procedure was done under general anesthesia (GA) in prone position that lasted four hours.

Postoperatively, the weakness improved in upper limbs and lower limbs. After the surgical procedure, the patient presented to the Ophthalmology Clinic of Princess Marina Hospital, with a complaint of visual loss of the right eye followed by left, of one week duration. Eye examination revealed light perception with sluggish pupillary reaction with deep anterior chamber, normal range of intraocular pressure, clear cornea and clear lens bilaterally. Fundal examination showed clear optic disc margin, no edema (cup to disc ratio- 0.4), normal macula, no retina or vitreous bleeding in both eyes. Computerized Tomography (CT) scan of the brain and eyes showed no space occupying lesion of the brain and no orbital swelling. Laboratory investigation such as Full blood count, ESR, HIV test, RPR for Syphilis, and serum angiotensin converting enzyme (ACE) were done which were normal.

The patient was treated with 40mg prednisolone tablet daily. After two weeks of steroid therapy, the visual acuity on the right eye was hand motion while on the left eye it was counting fingers at 3 meters. On the third week of his evaluation, the visual acuity of right eye and left eye were counting finger 3 meters and counting finger 5 meters respectively.

## Discussion

It is likely that a combination of hypotension, anemia, increased intraocular pressure secondary to being prone with head slightly dependent, long duration of surgery, significant intraoperative hydration all contributed to a reduction in perfusion pressure to the optic nerve intra operatively and resulted in the postoperative visual loss. Patients with preoperative risk factors (glaucoma, hypertension, diabetes, smoking and atherosclerosis) having prone spinal surgery at risk of severe blood loss are now known to be at significantly increased risk of postoperative visual loss secondary to ischemic optic neuropathy [1].

Recently it has been demonstrated that the prone position itself significantly increases intraocular pressure. A recent study in awake volunteers noted that there was a significant increase in intraocular pressure in all subjects when positioned prone [1]. Increased intraocular pressure can also be due to a number of factors including direct pressure on the globe and increased central venous pressure. Central venous pressure is increased by reduced venous return in the head-down position and obstruction to venous outflow if the head is turned to one side. Central venous pressure is also increased if there is direct pressure on the abdomen due to mal-positioning on an operating table compressing the abdomen and obstructing venous return to the heart. It has been demonstrated that, because of the absence of venous valves, changes in central venous pressure do translate into concomitant changes of ocular venous pressures and therefore can affect intraocular pressure [1].

It was also reported that major spine surgery can result in significant blood loss. Abdominal compression in the prone position can cause vena caval compression, reduction in venous return with resultant hypotension, venous stasis. Reduced intra-abdominal pressure is directly correlated with reduced blood loss during spine surgery. Positioning of patient in making the abdomen free from compression, maintaining or reducing intra-abdominal or bladder pressure are associated with reduced blood loss. Optimization of hemodynamics with maintenance of perfusion pressure of the optic nerve is recommended. Intraoperative blood loss can be reduced by careful positioning to avoid venous congestion at the surgical site, by meticulous surgical technique, by the use of anti-fibrinolytic agents. Induced hypotension is no longer recommended for patients having spine surgery the most important reason to avoid the use of induced hypotension is the potential for end-organ ischemia [2].

The use of both colloids and crystalloids for intravascular volume replacement, direct blood pressure monitoring, and assessment of volume status, monitoring hemoglobin periodically during cases with significant blood loss and optimization of hemodynamics with maintenance mean arterial pressure (MAP) within 20 to 25 percent of the preoperative baseline blood pressure is advised. Consideration should be given to a higher transfusion trigger in prone spinal patients at risk of postoperative visual loss. Assessing the postoperative vision in high-risk patients is crucial as soon as they are cooperative [2].

In patients operated with prone position for a significant length of time, it may be prudent to consider a 10 degree reverse Trendelenburg position to reduce the increased intraocular pressure during prolonged or position the head in a neutral position at or above the level of the heart [2]. Eyes should be checked throughout the case to avoid direct pressure on the globe. Use of the horseshoe headrest is not advised because of its close proximity to the eyes, the numerous reports of central retinal artery occlusion with its use, and the difficulty of checking the eyes for cervical spine procedures [2].

In this patient the absence of any finding in the optic disc and the retina, normal laboratory results and the normal CT scan, suggests that the most likely diagnosis is posterior ischemic optic neuropathy. Acute ischemic damage of the retro-bulbar portion of the optic nerve is characterized by abrupt, often severe visual loss; and initially normal-appearing optic discs. Posterior ischemic optic neuropathy (PION) is considered rare and is a diagnosis of exclusion. Perioperative PION has gained recent scrutiny because of the increasing number of reports that associate it with spinal surgery [3, 4].

There is no clear treatment for perioperative PION; vision loss does not typically improve significantly. Systemic high-dose steroid therapy has been used successfully in the management of non-trauma patients. However, other authors report no salvage of vision after steroid therapy, and thus steroid therapy for PION remains controversial [5].

Preventive strategies are of unproven benefit but are the only option to reduce the effect of this complication. Based upon the risk factors identified as noted above, the American Society of Anesthesiologists recommend that patients undergoing spine surgery undergo continuous hemodynamic monitoring and that colloids along with crystalloids be administered in patients who have substantial blood loss. Additional recommendations include that patients should be positioned to keep the head at or above heart level, with the neck in a neutral forward position when possible, and that staged rather than single, long-duration procedures should also be considered when appropriate. Vision should be assessed when the patient becomes alert [6–8].

The majority of patients with PION have immediate onset of visual loss, though in a small minority visual loss is delayed. In a case of our patient he started to notice visual reduction in the first one week after operation. This delay is hypothesized to be due to swelling of the ischemic neurons within the confines of the scleral canal. This swelling eventually impinges on the vascular supply and converts a reversible ischemic insult into irreversible axonal necrosis. This delayed presentation may occur anywhere from several hours to 10 days after the hypotensive event. The severity of visual deficit ranges from small field defects and diminished acuity to complete loss of light perception [8].

In our patient he presented with visual acuity of light perception for both eyes. His visual acuity improved following the use of Prednisolone tablet 40mg per day. The improvement of his vision could be either due to the effect of steroid treatment or may have occurred spontaneously.

## Conclusion

Visual loss needs to be considered as one of the post-operative complications with longer duration of spinal cord surgery. Preoperative identification of patients with risk factors, pre-operative patient counselling, intraoperative close monitoring and maintaining hemodynamic stability and proper positioning, and postoperative follow up of such cases is advised.
